# 34646 DEFICIENCY OF NOVEL ADIPOKINE TETRANECTIN INCREASES OBESITY AND INSULIN RESISTANCE IN FEMALES

**DOI:** 10.1017/cts.2021.453

**Published:** 2021-03-30

**Authors:** George Plasko, Sijia He, Jingjing Zhang, Fen Liu, Juli Bai, Lily Dong, Feng Liu

**Affiliations:** 1 UTHSA; 2 Central South University, Changsha, China

## Abstract

ABSTRACT IMPACT: Novel adipokines like tetranectin help explain why some people progress from obesity to diseases like diabetes, atherosclerosis, and dislipidemia OBJECTIVES/GOALS: Obesity has an established association with diabetes, dyslipidemia, and atherosclerosis. Preventing progression from obesity to insulin resistance requires understanding of the regulatory mechanisms involved in the loss of insulin sensitivity. Adipose tissue is well known to function as an endocrine organ that produces many kinds of adipokines. METHODS/STUDY POPULATION: Blood sample analysis from human patients and mice was used to determine associations between tetranectin and obesity. Samples were tested with a monoclonal anti-tetranectin antibody for detection with western blot. A tetranectin mutant knock out mouse line was compared to wild type littermates on high fat diet for 4 months. Insulin tolerance tests and glucose tolerance were used to determine progression to insulin resistance and glucose intolerance. Histological analysis of metabolic tissue was used to demonstrate adipocyte hypertrophy and liver steatosis. RESULTS/ANTICIPATED RESULTS: In the current study, we report the identification and initial characterization of a novel adipokine tetranectin. Tetranectin, which is coded by the C-type lectin domain family 3 member B (CLEC3B) gene, is ubiquitously expressed in various mouse tissues, whereas it is highly enriched in white adipose tissue. We found that the serum level of tetranectin was much higher in both obese and diabetic patients. Knocking out the tetranectin gene in mice protected against glucose intolerance in males but reduced insulin and glucose tolerance in females, without effects on food intake and body weight for either sex. Mechanistically, tetranectin targets liver tissues and its deficiency increases lipid accumulation in hepatocytes in females. DISCUSSION/SIGNIFICANCE OF FINDINGS: We have identified a novel adipokine which mediates a different metabolic crosstalk among tissues to maintain systemic glucose and lipid metabolism in different genders. Further investigation of tetranectin’s function could yield a new target for precise therapeutic treatment for obesity and its associated metabolic diseases in different genders

